# Targeted and responsive biomaterials in osteoarthritis

**DOI:** 10.7150/thno.78639

**Published:** 2023-01-16

**Authors:** Jiadong Li, Hao Zhang, Yafei Han, Yan Hu, Zhen Geng, Jiacan Su

**Affiliations:** 1 Institute of Translational Medicine, Shanghai University, Shanghai, 200444, China.; 2 Organoid Research Center, Shanghai University, Shanghai, 200444, China.; 3 School of Medicine, Shanghai University, Shanghai 200444, China.; 4 School of Life Sciences, Shanghai University, Shanghai 200444, China.

**Keywords:** Osteoarthritis, articular cartilage, targeted biomaterial, responsive biomaterial, drug delivery

## Abstract

Osteoarthritis (OA) is a degenerative disease characterized by loss of articular cartilage and chronic inflammation, involving multiple cellular dysfunctions and tissue lesions. The non-vascular environment and dense cartilage matrix in the joints tend to block drug penetration, resulting in low drug bioavailability. There is a desire to develop safer and more effective OA therapies to meet the challenges of an aging world population in the future. Biomaterials have achieved satisfactory results in improving drug targeting, prolonging the duration of action, and achieving precision therapy. This article reviews the current basic understanding of the pathological mechanisms and clinical treatment dilemmas of OA, summarizes and discusses the advances for different kinds of targeted and responsive biomaterials in OA, seeking to provide new perspectives for the treatment of OA. Subsequently, limitations and challenges in clinical translation and biosafety are analyzed to guide the development of future therapeutic strategies for OA. As the need for precision medicine rises over time, emerging multifunctional biomaterials based on tissue targeting and controlled release will become an irreplaceable part of OA management.

## Introduction

Osteoarthritis (OA) is one of the most common chronic degenerative joint diseases worldwide, often occurring in middle-aged and older adults over the age of 45 1. About 10% of men and 18% of women over 60 years old worldwide suffer from OA, and there is a gradual trend toward younger patients with OA due to the effects of obesity and strenuous exercise 2. The financial cost of an OA patient is estimated to be between $700 and $15,600 a year, placing a significant burden on both society and the individual 3. The pathological changes of OA mainly include wear and tear degeneration of articular cartilage, formation of bone fragments, synovial inflammation and subchondral bone remodeling 4. As the specific pathogenesis of OA is still unknown, the current clinical treatment of OA is mainly focused on relieving joint pain and delaying the development of OA 5-7. Currently, drug therapy, physical therapy and surgery are the primary treatments 8, 9. In the early stages of OA, pain relief and reduction of inflammation are achieved through appropriate exercise and weight loss and are supplemented with oral non-steroidal anti-inflammatory drugs (NSAIDs) 10. For patients with advanced OA, accompanied by severe articular cartilage wear, short-term symptomatic relief can be provided by intra-articular injections of hyaluronic acid (H) or glucocorticoids 11, 12. Due to the unique physiology of the joint cavity and the presence of synovial fluid, many drugs are characterized by poor water solubility, low cellular uptake, premature release or degradation. Targeted delivery and nonspecific release of drugs remain significant limitation to drug efficacy 13. A well-designed drug delivery system should have the appropriate size, biological barrier permeability, and proper drug release capacity 14-16. Therefore, many researchers hope to achieve OA treatment by developing novel and efficient drug carriers and delivery systems.

In recent times, many biomaterial carriers with cartilage or synovial targeting and responsiveness are emerging. Biomaterial carriers not only prolong the release of drug, but also exhibit higher tissue and cell specificity 17, 18. Among the various types of biomaterials, targeted and responsive biomaterials are more suitable for OA applications due to their small size and localizable delivery. They are often designed and assembled into functional structures to ensure accurate drug delivery 19-21. Currently, researchers developed various dual-functional biomaterials for OA treatment, such as cartilage-targeted combined enzyme/pH/ROS/NIR response 22-25, macrophage-targeted combined enzyme/ultrasound/pH response 26-28, and endothelial cell-targeted combined ultrasound response 29. Compared to passive cellular endocytosis, the introduction of chondrocyte-targeting peptides allows more efficient entry of responsive biomaterials into chondrocytes 22. Furthermore, Deng et al. designed a bifunctional biomaterial to target subchondral bone osteoclasts and synovial macrophages via RGD-αvβ3 integrin interactions 26. Subsequently, these biomaterials induced apoptosis by releasing celastrol in response to local MMP-9 26. Pro-inflammatory cell infiltration was found to be reduced and bone erosion improved in rats with advanced arthritis 26. Researchers have assembled engineered modified polymers into nanocarriers or hydrogels containing hydrophilic or hydrophobic regions loaded with drugs for targeted drug delivery or responsive drug release 13, 30. Targeted drug delivery is mainly divided into two types: passive targeting and active targeting. The former increases the local drug concentration by physical methods such as local injection, particle size reduction, and surface charge change, while the latter enhances targeted drug delivery by chemical methods such as modification of specific antibodies or affinity peptides on the carrier surface 31-33. In contrast, responsive biomaterials are usually stimulated by enzymes, reactive oxygen species (ROS), pH or external stimuli at the lesion site to release drugs, thus dramatically preventing the liberation and uptake of drugs in non-lesioned tissues 34.

In this review, we briefly overview the underlying physiopathology of OA and the currently used clinical treatments and their dilemmas. Then, we describe and summarize the characteristics of different types of targeted and responsive biomaterials and their research progress in OA. Finally, we discuss the potential limitations and challenges of biomaterials in the translation of clinical treatments for OA. We hope to provide further references and new perspectives for the future development of targeted and responsive biomaterials in OA therapy.

## Physiopathology of osteoarthritis

### Physiological properties of articular cartilage

The anatomy of articular cartilage is divided into hyaline cartilage, tidemark, and calcified cartilage 35. Articular cartilage transmits mechanical stress and aids in joint motion by providing an elastic and lubricated surface overlying the articular bone 36. It is a layer of avascular, nerve-free connective tissue composed of chondrocytes and extracellular matrix (ECM) 35, 37. Chondrocytes regulate the composition of the ECM through their own anabolism and catabolism, which constitute the most basic cellular units of cartilage homeostasis 38. ECM is composed of fibrin and proteoglycan, which has a certain mechanical strength and elasticity 36. Among them, supramolecular arrays with diameters between 25-400 nm are assembled from collagen types I, II, III, V and XI, which constitute the classical fibrous structure 39. Type II collagen, which accounts for 80% of the total collagen dry weight, is composed of three α1(II)-chains and has more glucosyl and galactosyl residues than other collagens so that they can interact with proteoglycans 39. The pore size between the cross-longitudinal collagen network is approximately 50-60 nm, which would allow small molecules of nutrients or drugs to disperse in the ECM and penetrate and act on the chondrocytes or subchondral bone 40. Proteoglycans consist of a number of core proteins attached to glycosaminoglycan (GAG) chains bonded non-covalently to long chains of hyaluronic acid, which give ECM its integral electronegativity, viscosity and resistance to compression 41. The dynamic stabilization of the relative ratios of collagen fibers and proteoglycans provides the extracellular environment that supports normal metabolism, proliferation, and differentiation of chondrocytes 42. Stimulated by mechanical stress or pro-inflammatory factors, anomalous higher expression of matrix metalloproteinases (MMPs) disrupts the dynamic balance in the ECM, which ultimately leads to chondrocyte apoptosis and articular cartilage degradation, thus further contributing to the progression of OA 43.

### Pathogenesis and pathological changes of osteoarthritis

**Figure 1:**
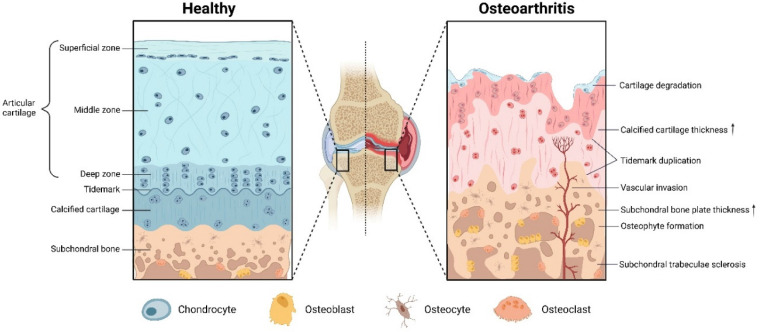
The physiological structure of articular cartilage and pathological changes of OA. Created with BioRender.com.

According to the causative factors, OA can be broadly divided into primary OA caused by gene, age and gender factors, and secondary OA caused by sports trauma, metabolic disorders, obesity, etc. 44, 45. The latest data show that about 240 million people worldwide are afflicted by OA 2. Among the aging population over 60 years old, women (18%) are more likely to suffer from OA than men (10%) 2. In England, 53.23% of respondents over the age of 50 reported that they suffer from OA-related pain in one or more joints 2. The pathological changes of OA involve the entire joint including the synovial membrane and subchondral bone (Figure 1). First, the articular cartilage becomes softened and inelastic, and the cartilage layer velvety erupts and exposes the subchondral bone plate 4, 46. Secondly, abnormal ossification of the cartilage at the edge of the joint forms an osteoid with neurovascular invasion and causes joint pain 4. Subsequently, vascularization at the edge of the cartilage or at the tendon attachment forms a bony mass via cartilage ossification, and this causes joint pain, stiffness, deformity and dysfunction 4. Finally, collateral synovial inflammation and subchondral bone osteophytes will lead to macrophage infiltration and neurovascular invasion, further exacerbating the OA process 4.

At the molecular level, Interleukin-1β (IL-1β), produced by chondrocytes, osteoblasts, synoviocytes and macrophages, is considered to be the most critical inflammatory factor in the pathogenesis of OA, which can act independently or synergistically with other cytokines to cause articular cartilage degradation and joint inflammatory responses 47, 48. IL-1β increases nitric oxide (NO) and prostaglandin E2 (PGE2) expression by activating its downstream transcription factors, triggering synovial vasodilatation hyperplasia and joint cavity pain 49. At the same time, the autocrine production of tumor necrosis factor-α (TNF-α) and Interleukin-6 (IL-6) by the above cells is stimulated, synergistically enhancing the pro-inflammatory effect of IL-1β and producing proteases such as the MMPs family and a disintegrin and metalloproteinase with thrombospondin motifs (ADAMTS) family to cleave and disassemble type II collagen and aggrecan in the ECM, thereby destroying cartilage structure 47, 48, 50. Furthermore, because ADAMTS plays a critical role in overall tissue renewal, elevated ADAMTS expression is not only associated with joint degeneration and deterioration, but may also reflect the persistence of OA inflammation and injury 51. Some scholars have also suggested that alterations in the subchondral bone microenvironment precede the occurrence of cartilage destruction, where abnormal mechanical loading and activation of TGF-β in bone-chondral crosstalk are essential factors leading to anomalous bone remodeling, angiogenesis, and sensory innervation 4. Although numerous studies are available to explain the pathogenesis of OA at the cellular and molecular levels, a substantial body of research combined with clinical data is still needed to fully understand the underlying causative factors of OA.

### Clinical treatment of osteoarthritis and its dilemma

The clinical treatment of OA is divided into two main categories: medication and surgery, while medication is split into two groups: symptom relief and condition improvement 7. NSAIDs are the most widely used drugs that diminish the production of inflammatory factors and exert analgesic effects 52. However, NSAIDs have gastrointestinal, neurological, hematologic and allergic side effects, as well as symptomatic treatment and relief of patient suffering, but not slowing down the progression of the disease 53, 54. Another group of drugs can significantly relieve the clinical symptoms of OA is glucocorticoids, which have a potent anti-inflammatory effect and can suppress the proliferation of aberrant tissues and reduce joint effusion, thus achieving a significant effect of detumescence and analgesia 55. However, long-term use of glucocorticoids leads to bone loss and accelerates articular cartilage and subchondral bone lesions, which limits their clinical application and eliminates them as a routine treatment option 56. HA is an integral component of the joint fluid and cartilage matrix, commonly used in intra-articular injection for the treatment of OA 57. HA could restore the viscoelasticity of joint fluid, enhance lubrication and shock absorption cushioning, reduce joint friction, relieve pain, prevent cartilage degradation, and lower the production of MMPs, which has significant efficacy in clinical practice 12, 58. Nevertheless, joint cavity injection of HA has high demands on operation technique and operation environment, and may cause short-term complications such as joint edema and infection, so there are still plenty of questions and controversies concerning the use of HA 59. Glucosamine and chondroitin sulfate (CS) suppress the activity of proteases and degradative enzymes, which also target articular cartilage, making them ideal drugs for improving OA 60.

In advanced OA, severe joint deformities will occur, rendering pharmacological treatment futile, and surgical treatment is the only option, most commonly with arthroscopic debridement and total joint replacement 61. Arthroscopic debridement is indicated for mild to moderate OA and often provides a more satisfactory outcome 62. Total joint replacement, on the other hand, is suitable for patients with severe OA and can not only significantly reduce pain, but also correct joint deformities and restore joint function, making it the last choice for all OA patients 63. Although surgery can achieve effective treatment, it is expensive and may lead to serious postoperative complications and medical risks 64. For example, patients undergoing total knee arthroplasty have twice or more the incidence and mortality of venous thromboembolism due to postoperative complications such as coagulation disorders, electrolyte disturbances, and pulmonary complications 64, 65. In addition, the recruitment of bone marrow cells by microfracture to form a smooth and strong repair tissue can also replace the role of cartilage 66. The recovery from microfracture surgery is directly related to the patient's own ability to repair 66. Significant efficacy in young, low-weight patients, but poor efficacy in older, obese patients. After surgery, patients need to recover for 12 months or more with the assistance of a stent 67, 68. With the maturation of *in vitro* culture techniques, autologous chondrocyte implantation (ACI) is better able to regenerate normal articular cartilage structures 68. ACI allows for the implantation of mature and active hyaline cartilage in a single operation, while avoiding immune rejection 68. But ACI technology is still in its infancy, and lab-cultured chondrocytes often mean higher treatment costs 69, 70. Collectively, owing to the drawbacks and limitations of existing clinical treatment strategies, there is an imminent demand for the development of effective, versatile, targeted, and responsive OA therapies.

## Responsive biomaterials in osteoarthritis

Currently available anti-inflammatory and analgesic drugs, either orally or by intra-articular injection, suffer from poor water solubility, low cellular uptake, poor drug concentration distribution or premature degradation 71. By focusing on the responsiveness of biomaterials to different substrates or external stimuli, researchers are developing responsive biomaterials that can release encapsulated drugs efficiently and accurately 34, 72. These responsive biomaterials can minimize early or abrupt release behavior to address the problems of low bioavailability and low efficacy of anti-OA drugs. In this section, the advances in research on enzymes, ROS, pH responsive and external stimulus responsive biomaterials in the treatment of OA are reviewed (Table 1).

### Enzyme-responsive biomaterials

Since enzymes possess a high degree of selectivity and efficiency, drug release in a variety of ways such as surface ligand activation and chemical bond breaking can be achieved by introducing functional groups sensitive to enzymes aberrantly expressed in the OA microenvironment into biomaterials 99. With a high secretion of multiple matrix-degrading enzymes and degradation-activating enzymes in OA, enzyme-responsive biomaterials are designed to control drug release by exploiting the differences in enzyme activity in tissues 100. Namely, when administered early in OA or prophylactically, the drug is released in low or no volume to avoid side effects, while a large volume of drug can be unleashed swiftly in advanced disease. Enzyme-responsive wise biomaterials often have enzyme-sensitive backbones or biodegradable ester bonds as linkages, resulting in efficient controlled release behavior 71.

**Figure 2:**
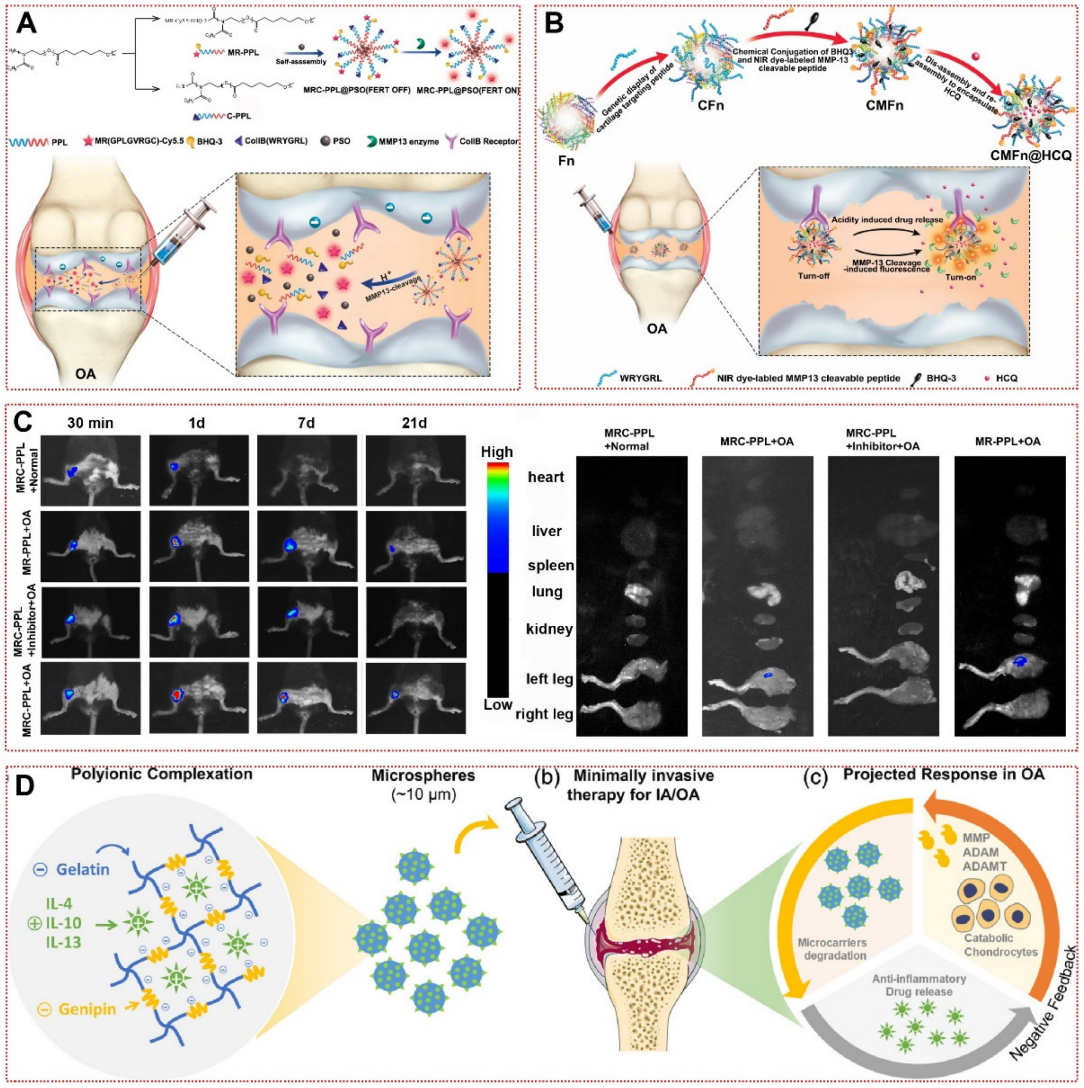
Enzyme-responsive biomaterials for OA treatment: **(A)** Schematic illustration of the synthesis and working mechanism of MMP-13 and pH responsive theranostic MRC-PPL@PSO nano-micelles for osteoarthritis 22. High expression of MMP-13 in OA joints generates fluorescent signals by cleaving sensitive peptides to release Cy5.5. In addition, the low pH environment moderates the release of PSO in micelles to treat cartilage inflammation. Copyright 2020, BMC. **(B)** Schematic illustration of the CMFn@HCQ as a cartilage-targeting and MMP-13/pH dual-stimuli activatable theranostic nanoprobe for *in vivo* MMP-13 imaging and precision therapy of osteoarthritis 73. Ferritin in the CMFn@HCQ nanocage dissociates in an acidic environment to release HCQ. Simultaneously, MMP-13 severs the short peptide that connects the burster and the free NIR dye can visualize the concentration of disease progressing MMP-13 in OA. Copyright 2019, Elsevier. **(C)** *In vivo* fluorescence imaging of the major organs and bilateral joints 21 days after MRC-PPL joint cavity injection 22. Copyright 2020, BMC. **(D)** Bioresponsive microspheres for delivery of the anti-inflammatory cytokines in osteoarthritis 75. Gelatin microspheres are degraded by the rate of catabolic factors produced by OA microenvironment cells to release IL-4, IL-10 and IL-13 to reduce chondrocyte inflammation. Copyright 2019, Wiley.

Although enzymes have received extensive attention in studies on their application as triggers in malignancies and cardiovascular diseases, their application in OA therapy is still in its infancy so far 101. MMPs, one of the key enzymes in the deterioration of OA, are commonly manufactured by chondrocytes under inflammatory conditions and lead to cartilage erosion by breaking down type II collagen and aggrecan in the joint matrix 43. Out of this inspiration, Lan et al. created an MMP-13 responsive therapeutic integrated nanoplatform by modifying specific peptide substrates of MMP-13 22. By respective coupling type II collagen-binding peptide and Cy5.5-labeled MMP-13-responsive peptide substrate to poly(2-ethyl-2-oxazoline)-poly(ε-caprolactone) (MRC-PPL), self-assembling and loading the Chinese herbal active ingredient psoralidin (PSO), the multifunctional MRC-PPL@PSO achieved the release of therapeutic drugs while being able to report OA via fluorescent signals (Figure 2A) 22. The fluorescent signal of MRC-PPL micelles in healthy joints or MMP-13 inhibitor-treated OA joints was significantly lower than in the OA group, representing that MRC-PPL micelles were mediated by MMP-13 activity in response to the onset of OA joints (Figure 2C) 22. Similarly, Chen et al. achieved responsiveness to altered MMP-13 expression in OA by loading hydroxychloroquine with MMP-13-responsive ferritin nanocages (CMFn@HCQ) in an almost identical manner to the former (Figure 2B) 73. This shows the promise of multifunctional responsive biomaterials for image-guided OA precision therapy. In addition to MMP-13, synovial inflammation and subchondral bone lesions in OA are often accompanied by MMP-2/MMP-9-mediated matrix remodeling and angiogenesis 102. Enzyme-responsive hydrogel loaded with triamcinolone acetonide (TA) have favorable biocompatibility. When it was incubated in PBS at 37°C or in synovial fluid without MMPs for 30 days, only 20% of the drug was released 74. The addition of MMP-2/MMP-9 or inflammatory synovial fluid resulted in a sustained release of TA over 30 days, which was reversed by the addition of MMPs inhibitors 74. Furthermore, responsive biomaterials using osteoblasts or vascular endothelial cells highly expressing MMP-2/MMP-9 as enzymatic response targets might be a new strategy to treat OA to restore subchondral bone structure and mechanical stress disorders 103, 104. More interestingly, gelatin microspheres delivering anti-inflammatory cytokines in response to collagenase produced by inflammatory cells were able to effectively restrict IL-1β or LPS-induced NO production, which may further validate the promising application of responsive biomaterials in OA (Figure 2D) 75.

### ROS-responsive biomaterials

ROS broadly refers to oxygen-derived radicals and non-radicals, and contains superoxide anions (O^2-^), hydrogen peroxide (H_2_O_2_), hydroxyl radicals (OH**·**), nitric oxide radicals (NO**·**) and singlet oxygen (^1^O_2_), which have high chemical reactivity because they contain unpaired electrons 38. ROS under physiological conditions is primarily generated by the electron transport chain of mitochondria, which in physiological concentrations facilitate immune defense, apoptosis regulation, and intracellular signal transduction 105. After the injury occurrence, the intracellular or microenvironmental antioxidants such as glutathione (GSH) and superoxide dismutase (SOD) are insufficient to eliminate the excessive production of ROS, which will trigger intracellular metabolic disorders and biomolecular damages, eventually leading to apoptosis 105, 106. Recent studies have shown that ROS levels are significantly increased in the joints of OA patients, moreover, it has been shown that ROS clearance can be effective in relieving OA 105, 107, 108. Oxidative stress generated by excessive ROS in the joint will lead to chondrocyte membrane disruption and synthesis of proteoglycan matrix 109. ROS-induced activation of the NF-κB pathway stalled chondrocyte growth and secreted MMPs, exacerbating cartilage inflammation 105. With the progression of aging, chondrocytes will become increasingly sensitive to ROS damage, inducing more cellular senescence and apoptosis 110. Such evidence reveals that abnormally elevated ROS is inextricably linked to OA pathological progression, suggesting that utilizing high levels of ROS in the OA tissue microenvironment is a promising trigger for responsive drug release 110.

**Figure 3:**
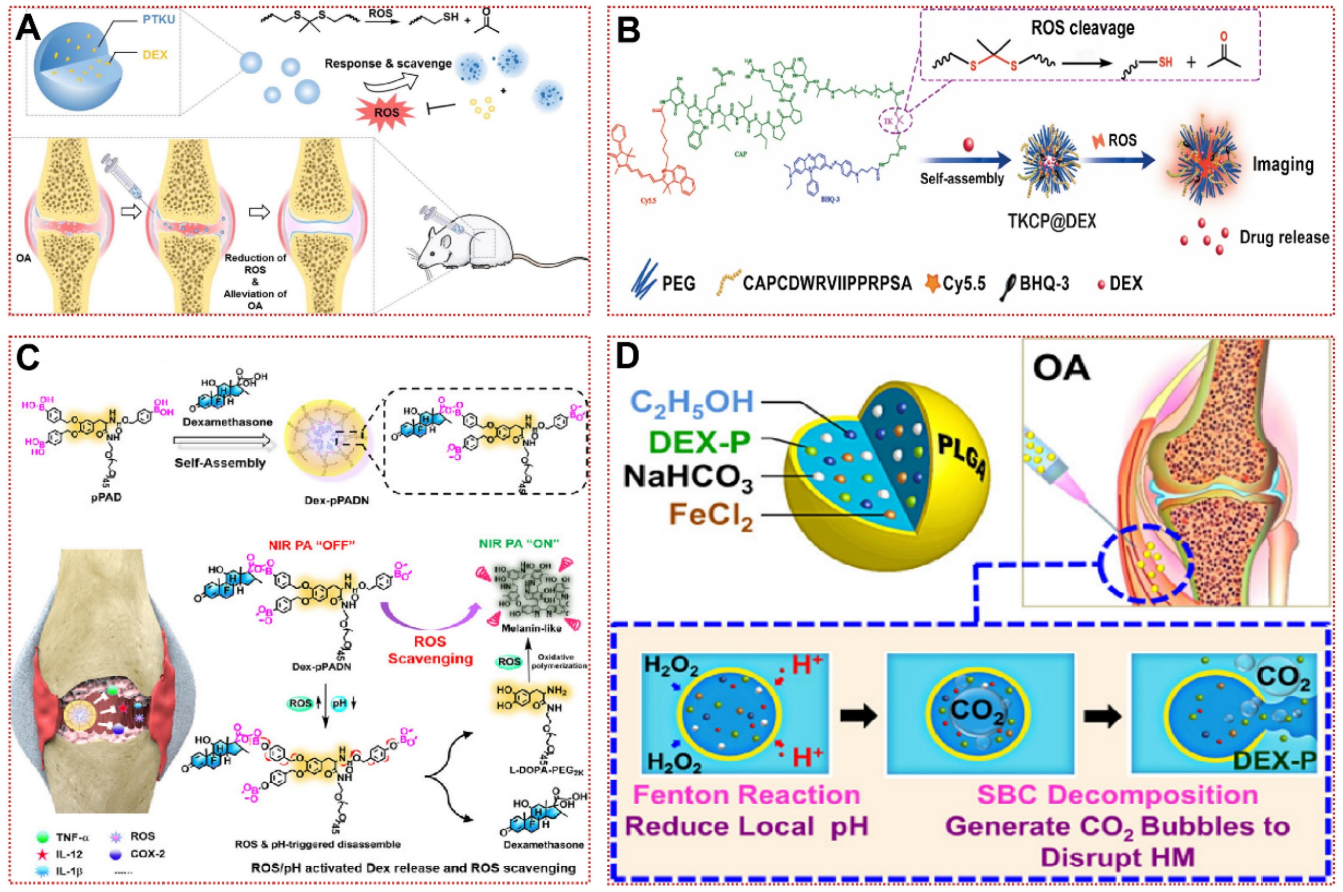
ROS-responsive biomaterials for OA treatment:** (A)** Schematic illustration of the ROS scavenging/responsive mechanism of the thioketal repeating unit in PTKU@DEX NPs for OA treatment 76. Copyright 2021, Elsevier. **(B)** Schematic illustration of the self-assembly of ROS-responsive nanoparticles for bioimaging and targeted therapy of osteoarthritis *in vivo* 24. Copyright 2021, BMC. **(C)** Schematic illustration of the preparation of Dex-pPADN for the treatment of osteoarthritis 77. Copyright 2021, Wiley. **(D)** Composition/structure of the ultrasensitive ROS-responsive gas-generating HM developed herein and its mechanism in the treatment of OA 78. Copyright 2015, American Chemical Society.

The mechanisms of ROS-responsive biomaterials are divided into two types: chemical bond breaking (e.g., polythioketal, polyproline, phenylboronic acid, and ester-containing polymers) and hydrophilic-hydrophobic conversion (e.g., polypropylene sulfide, thioether-containing polymers, monotellurium polymers, and monoselenium polymers) 111. Zhang et al. used polythioketal (PTK) as a soft linkage, 1,4-butanediol as a chain extender, and Hexamethylene Diisocyanate (HDI) as a hard linkage to synthesize a ROS-responsive polyurethane (PTKU) with superior mechanical properties (Figure 3A) 76. Compared to the control, PTKU NPs underwent a dramatic morphological transformation after incubation with OH**·** (1000 mM), and all thioketal bonds were broken, leading to nanoparticle depolymerization and structural relaxation 76. *In vivo* experiments, PTKU NPs not only demonstrated favorable articular targeting, but also boosted the shift of synovial macrophages to M2 type and downregulate the expression of MMP-2 and various inflammatory factors in articular cartilage 76. Similarly, TKCP@Dex NPs could also be obtained by encapsulating dexamethasone (Dex) with thioketal (TK) and cartilage-targeting peptide (CAPDWRVIIPPRPSA) functionalized PEG (Figure 3B) 24. Under the *in vitro* catalysis of KO_2_, TKCP@Dex NPs progressively broke down the thioketone bond to release Cy5.5 and Dex to visualize the treatment, scavenging IL-6 and MMP-13 without causing cytotoxicity 24. In the monosodium iodoacetate (MIA)-induced OA model, high concentrations of ROS in the joint facilitated aggregation of the nanoprobe and rapidly peaked within 24 h 24. H&E and safranin O-fast green staining revealed a better recovery of the cartilage matrix than Dex injection, demonstrating the effectiveness of the vector in the organism 24. Zhao et al. achieved high loading and controlled release of Dex by assembling L-dopamine modified with phenylboronic acid into nanoparticles (Dex-pPADN) in aqueous solution 77. Through a cascade of redox reactions triggered by ROS at the site of inflammatory damage, Dex-pPADN formed melanin-like structures and exhibited extremely strong anti-inflammatory and antioxidant effects (Figure 3C) 77. Due to the structural transformation after oxidation by ROS, Dex-pPADN was available as a contrast agent to detect the progression of OA noninvasively, with luminescence intensity proportional to the severity of OA 77.

Compared with the previous study, the water-in-oil-in-water biphasic hollow microspheres (HM) composed of ethanol, ferrous chloride, Dex, and sodium bicarbonate achieved ultra-sensitive ROS responsiveness as shown in Figure 3D 78. The low concentration of H_2_O_2_ passes through the PLAG shell and then oxidizes ethanol to acetic acid by the Fenton reaction with Fe^2+^ catalysis, subsequently sodium bicarbonate decomposes in an acidic environment to produce abundant carbon dioxide (CO_2_), which eventually leads to the collapse of HM 78. As shown in the SEM images, HM is uniformly dispersed hollow spheres and form large pores at 50 μM H_2_O_2_/pH 6.8, making this unique structure ideal for the release of cargo 78. Nevertheless, researchers should be mindful of the potential toxicity of these responsive biomaterials with excessive local Dex concentrations. In addition, tannic acid and tetrahydroxydiboron have been reported to form borates with ROS response and NO scavenging ability 79. In conclusion, ROS-responsive biomaterials hold exciting promise for the treatment of inflammatory diseases not restricted to OA.

### pH-responsive biomaterials

In OA lesioned joints, synovial fluid pH decreases from 7.4 to 6.0 owing to local tissue hypoxia metabolism and acidosis 112, 113. Even in different areas of the lesion, the pH of the joint tissue reflects some variability 114. Articular cartilage (6.3) had a slightly lower pH than the meniscus (6.5) 114. This significant pH shift in the pathological microenvironment is considered as an ideal response condition to modulate drug delivery 115, 116. Most pH-responsive nanomaterials are based on chemical bond breakage of cleavable groups or protonated dissociation of ionizable groups, leading to carrier acidolysis, representative of which are esters, Schiff bases, amides, vinyl ethers, and imines 34. To overcome the high lipophilicity limitation of the anti-inflammatory drug andrographolide (AG), He et al. formulated a ph-responsive biomaterial (AG@MSNs-PAA) based on polyacrylic acid (PAA) 80. Since PAA degrades in an acidic environment, the progressive release of AG over 72 h reversed IL-1β-induced chondrocyte apoptosis, showing recovery of Col2α1 and Aggrecan 80. Beyond polymers, metal-organic frameworks (MOF) and inorganic materials also hold promising promise for the development of pH-responsive biomaterials 81, 82. Xiong et al. synthesized an acid-dissolvable biomaterial MOF@HA@PCA by HA modification and loading with protocatechuic acid (PCA) 81. The release of cargoes in MOF@HA@PCA showed “fast followed by slow” pattern, with a 1.6-fold increase in PCA release rate in PBS at pH=5.6 relative to pH=7.4 81. MOF@HA@PCA demonstrated a favorable safety and potent anti-inflammatory effect, substantially downregulating the expression of inflammatory markers including MMP-3, MMP-13, COX2, and iNos 81. More interestingly, the bisphosphonate-conjugated nano-apatite (NP-BP) exhibited distinct release behaviors at different pH 82. NP-BP targets overactive osteoclasts in subchondral bone to reverse cartilage fibrosis and angiogenesis, reflecting a novel therapeutic strategy for OA 82.

**Figure 4:**
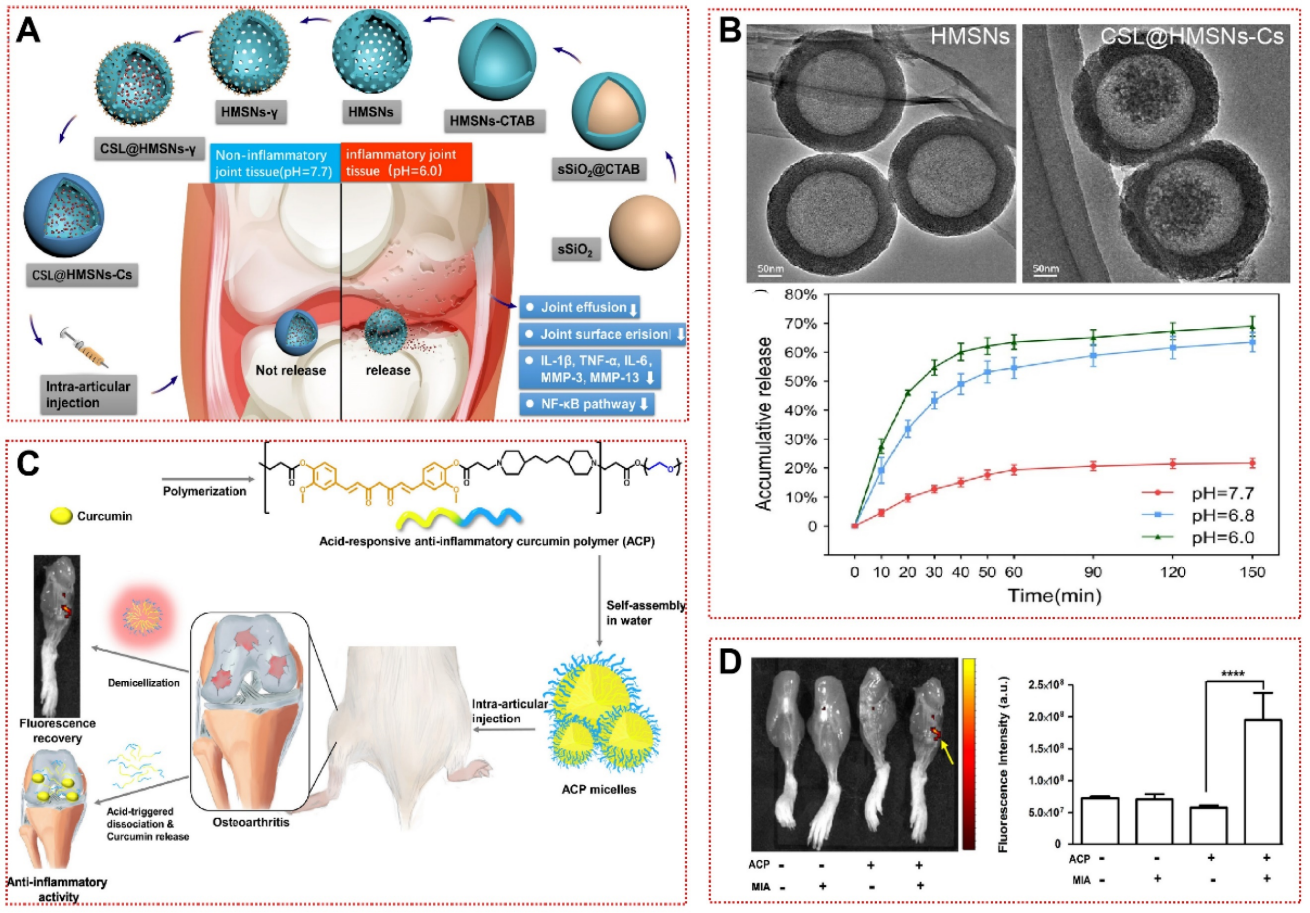
pH-responsive biomaterials for OA treatment: **(A)** Schematic assembly procedure of CSL@HMSNs-Cs and mechanism of intra-articular injection of CSL@HMSNs-Cs serving as a pH-responsive medicine for anti-inflammatory therapy of osteoarthritis 85. Copyright 2020, BMC. **(B)** TEM images of HMSN, CSL@HMSNs-CS and cumulative release curves of CSL@HMSNs-CS in different pH PBS 85. Copyright 2020, BMC. **(C)** Schematic showing pH-responsive polymeric prodrug of curcumin as a therapeutic system for osteoarthritis 84. Copyright 2019, Elsevier. **(D)** Fluorescence images and quantitative analysis of MIA joints injected with ACP micelles 84. Copyright 2019, Elsevier.

Additionally, some strategies are based on pH-dependent hydrophobic alterations or internal gas generation, allowing for overall morphological structural changes to achieve on-demand drug release 83-85. Jin et al. constructed chitosan-coated hollow mesoporous silica nanoparticles (CSL@HMSNs-CS) loaded with poorly hydrosoluble Celastrol (CSL, a pentacyclic triterpene compound) 85. The hollow structure (HMSN) was first prepared by hydrothermal etching, followed by anchoring the chitosan to the HMSN surface by cross-linking agent to enclose the CSL in the cavity inside the sphere (Figure 4A and 4B) 85. When H^+^ is present at high levels, the protonation of free NH_2_ in Cs leads to increased water solubility and rupture of the protective layer, triggering pH-responsive cargo release 117. CSL@HMSNs-CS significantly reduced IL-1β and TNF-α in chondrocyte culture supernatants by inhibiting the NF-κB signaling pathway, as well as alleviating pain behavior in OA 85. In the MIA-induced OA model, poly(β-amino ester) (PAE) amphiphilic polymers released curcumin in a pH-sensitive manner, demonstrating superior cartilage matrix reconstitution than the equivalent curcumin (Figure 4C) 84. ACP micelles will aggregate more significantly in OA joints (Figure 4D). Hu et al. synthesized a responsive biomaterial with carbonates for the treatment of OA 83. Protons in the acidic microenvironment could penetrate the PLGA shell to produce CO_2_ with NH_4_HCO_3_ to lead to lactate disintegration, then Rh-PLGA-NPs@NH_4_ released three times as much rhein (Rh) as the control in the OA synovial fluid mimic 83. In future applications, we must consider the possible safety hazards associated with these degradation products and improve the sensitivity of biomaterials to environmental pH changes to adapt to the needs of different environments in OA therapy.

### Stimulus-responsive biomaterials

In contrast to endogenous factors like enzymes, ROS, and pH, simple and manipulable exogenous stimuli have also been used to initiate the release of loaded drugs 118, 119. Operators can modulate the release behavior of responsive biomaterials by applying light, thermal, magnetic or ultrasound to the lesion site 120. Moreover, as a high-precision drug delivery system, stimulus-responsive biomaterials can be used to add or remove external stimuli at will, or to achieve simultaneous release of multiple sites, as required, ultimately enabling individualized OA treatment modalities (continuous or intermittent) 121. Thus, all these strengths offer great possibilities for the application of stimulus-responsive biomaterials in OA therapeutics.

**Figure 5:**
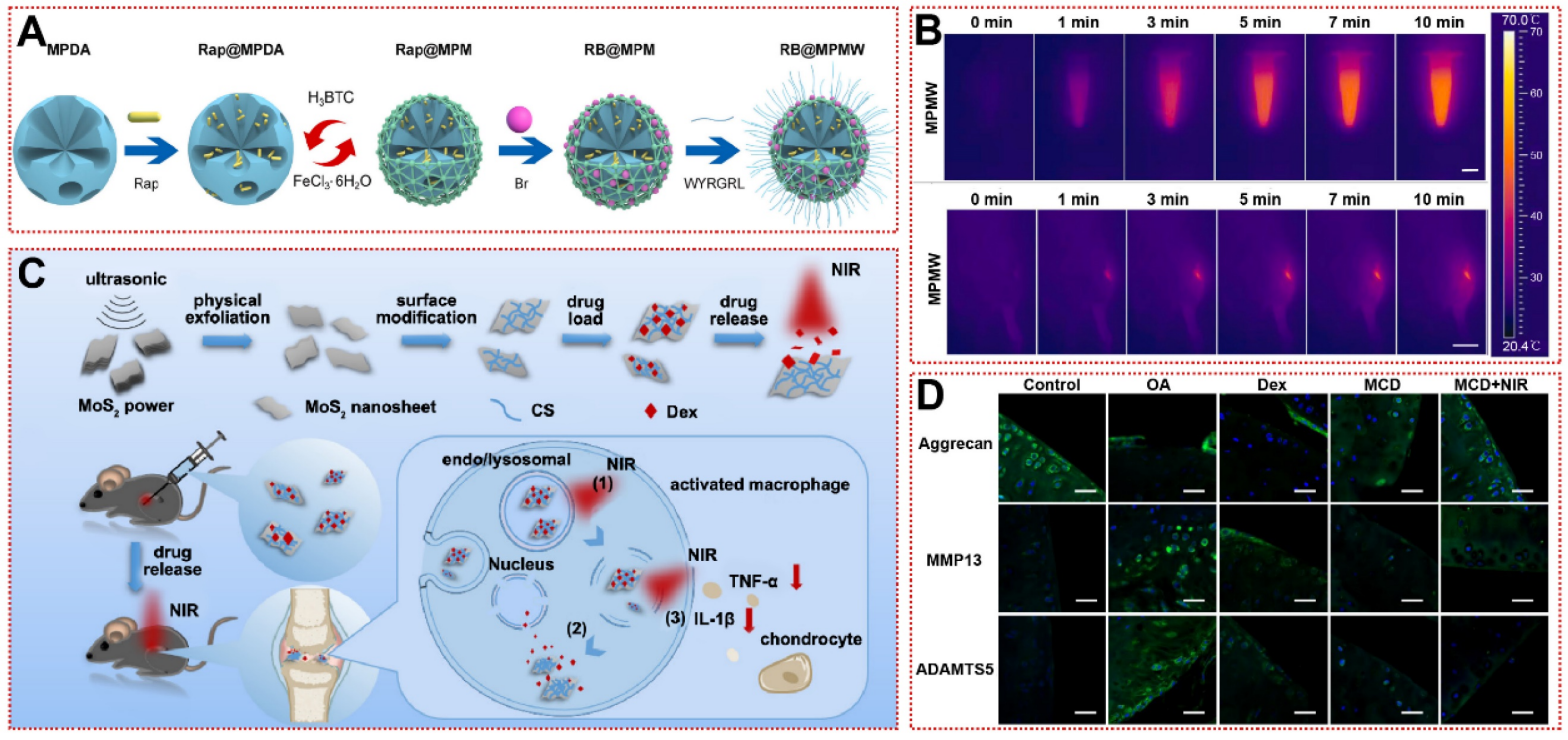
Stimulus-responsive biomaterials for OA treatment: **(A)** The schematic diagram of fabrication of RB@MPMW 25. Copyright 2021, Elsevier. **(B)** *In vitro* and *in vivo* photothermal performance of MPMW under 808 nm NIR laser irradiation at different times 25. Copyright 2021, Elsevier.** (C)** Synthesis process of the drug-loaded nanosystem and its drug release in response to NIR light *in vitro* 88. Copyright 2019, American Chemical Society. **(D)** Immunohistochemical fluorescence staining of articular cartilage matrix markers (Aggrecan) and cartilage inflammation markers (MMP13, ADAMTS5) in mice after 28 days of treatment with Dex-loaded molybdenum disulfide nanosheets 88. Copyright 2019, American Chemical Society.

After the onset of inflammation, clinical practice reveals swelling of the knee joint accompanied by an increase in temperature of 2-3°C 34. Kang et al. reported a thermo-responsive nanosphere that can independently release kartogenin (KGN) and diclofenac (DCF) at different rates in response to variations in temperature 86. The nanospheres consist of a cross-linked network of amphiphilic tri-block polyoxyethylene, pluronic F127 and chitosan loaded with KGN on the outside, wrapped with polyoxypropylene and a pluronic F127 core loaded with DCF 86. Nanospheres not only promote chondrogenesis of hBMSC *in vitro*, but also rescue cartilage matrix deficiency and abnormal ossification (e.g. osteophyte, bone spurs) *in vivo* 86. The inverse opal hydrogel scaffold was modified with poly(N-isopropylacrylamide) to acquire temperature responsiveness, releasing diclofenac sodium only in response to inflammation or exercise fever, which greatly enhances drug utilization 87. But endogenous heat is not sufficient for high-precision and spatiotemporally controllable drug release, limiting the clinical translation of thermo-responsive biomaterials. Considering that near-infrared (NIR, λ=780~1100 nm) light has superior tissue penetration (5~10 mm), researchers have developed photothermal therapy (PTT) with specific wavelengths of laser as the arouser 122-124. Xue et al. constructed a MOF-based mesoporous polydopamine (PDA) multifunctional targeting nanocarrier (RB@MPMW) loaded with rapamycin (Rap) and bilirubin (Br) for the first time (Figure 5A) 25. Whether in articular cavity or aqueous solution, RB@MPMW exhibited outstanding photothermal properties, reaching over 40°C within 3 min (Figure 5B) 25. Meanwhile, under NIR irradiation, Rap and Br released by RB@MPMW intensely suppressed P65 phosphorylation and mitochondrial dysfunction, suggesting the restoration of chondrogenic anabolism 25. In an anterior cruciate ligament transection (ACLT) rat model, the RB@MPMW group had the lowest OARSI score and LC3B autophagy-positive cell count, confirming the efficacy of NIR-responsive biomaterials in OA 25. Similarly, Dex-loaded molybdenum disulfide nanosheets diminished macrophage-derived TNF-α and IL-1β by NIR exposure that rescued joint surface erosion and bradykinesia in OA mice (Figure 5C) 88. Immunofluorescence showed that cartilage lesion caused by OA was reversed (Figure 5D) 88.

Magnetic manipulation has attracted interest due to its biocompatibility and non-invasive operation 125. It is a magnetically driven manipulation achieved by introducing magnetic nanoparticles or magnetic fields. In the presence of an alternating magnetic field, the magnetic nanoparticle transit rate in bovine articular cartilage was increased by nearly 50-fold 89. Magnetic nanoparticles are often made of trivalent iron complexes. After chitosan modification, meloxicam achieves an encapsulation rate of 82% 90. In addition, nanoparticles with a gold-iron-gold shell structure coupled PPT and *in vivo* MRI, enabling the integration of diagnosis and treatment 91. The methotrexate loaded in the core is precisely released in the inflamed joint by an external magnetic field 91, 92. Superparamagnetic iron oxide particles integrated diagnosis and therapy under an applied magnetic field. It carries anti-inflammatory drugs to combat local inflammation while enhancing magnetic resonance imaging sensitivity 93, 125. Magnetic nanoparticles with a superparamagnetic iron oxide nanoparticle core were used for responsive delivery of active siRNA to rat joints 94. Magnetically responsive nanoparticles improved siRNA stabilization and macrophage uptake 94. The subsequently released siRNA inhibited IL-2/IL-15Rβ expression to alleviate joint inflammation 94. In addition, the addition of dynamic magnetic field is more favorable for the chondrogenic differentiation of BMSC on the surface of biomaterials 95. All of these results demonstrate the feasibility and efficiency of magnetically responsive biomaterials for OA therapy.

Ultrasound-responsive biomaterials for arthritis treatment are being developed in recent years. Liao et al. successfully delivered diclofenac sodium to rat joints by ultrasound responsiveness 96. This transdermal delivery reduced angiogenesis and joint swelling in the inflamed area 96. Under safe low frequency ultrasound stimulation, ultrasound-responsive liposomes are enriched in the joint and release methotrexate to inhibit arthritis progression 29. Ultrasound turns drug-loaded liposomes into “nanobombs” by locally triggering the rupture of the liposomes 27, 97. Under folic acid-mediated targeted delivery, the uptake of nanobombs by activated macrophages is increased 27, 97. Subsequently, localized and precise release of Dex alleviated inflammatory cell infiltration and cartilage destruction in the joint cavity under ultrasound stimulation 27, 97. Alternatively, ultrasound responsiveness of biomaterials can be conferred by introducing sonosensitizer sparfloxacin into the structure 98. A recent study demonstrated that ultrasound-activated H_2_O_2_ nanoenzymes produce O_2_ to alleviate hypoxia in the joint cavity, thereby downregulating hypoxia-inducible factors to prevent angiogenesis 98. Unfortunately, the biggest disadvantage of ultrasound is that it is strongly attenuated by the dense bone. The lack of sufficient penetration may also be an important reason limiting the use of ultrasound-responsive biomaterials in bone-related fields. Overall, the application of stimulus-responsive biomaterials for OA therapy is still in the infancy of simple assembly of material precursors and drugs. More systematic and comprehensive preclinical studies are needed in the future to confirm their reliability and stability.

## Targeted biomaterials in osteoarthritis

In the treatment of OA, whether by intra-articular injection or systemic injection, the disorganized diffusion of the drug agent in the organism is fundamental to the efficacy of the drug 126. The diminished efficacy and low utilization of the drug brought about by this disorganized proliferation forced physicians to raise the dose, creating a vicious cycle that ultimately leads to side effects 126. Therefore, the targeted delivery of drugs by designing reliable OA targeting strategies has become a hot topic of recent research. Targeted biomaterials not only expand the therapeutic window of the drug, but also enhance penetration and retention time 127, 128. Next, we introduce various passive and active targeting strategies in the hope of achieving the healing of OA through targeted drug delivery to cartilage or synovium (Table 2).

### Passive targeting strategy

As an avascular dense connective tissue, the cross-linked network formed by type II collagen with a pore size of about 50-60 nm and aggrecan with a chain spacing of about 20 nm hinders the uptake of most drugs 152-154. Many promising experimental drugs have failed in clinical practice due to the fact that they remain on the surface of the cartilage and do not penetrate into the interior and subchondral bone 155-157. The pore size and porosity of the cartilage matrix also vary with the progression of OA, and either too large or too small a particle size will result in poor penetration 158, 159. Thus, suitable and homogeneous particle size will be a shortcut to increasing the effective dose and penetration of the drug. Several studies have shown that morphologically intact healthy cartilage only allows particles below 10 nm to enter the deeper layers, while 15 nm particles remain in the cartilage surface after 24 h 160, 161. However, during the progression of OA, ECM will allow the entry of nanoparticles up to 300 nm, which may be due to the disruption of the cartilage matrix 135, 147. Endocytosis of 15 nm MnO_2_ nanoparticles by chondrocytes was observed at 30 μm after 24 h incubation with bovine cartilage explants 130. Furthermore, the penetration of nanoparticles in the synovium is similarly size-dependent. Only 5-nm gold nanoparticles successfully penetrated and would reduce synovial inflammatory biomarker concentrations, corroborating the importance of particle size in OA treatment 129. Overall, smaller particle size tends to exhibit higher cartilage penetration and cellular uptake, which is one of the important bases for achieving OA targeting.

**Figure 6:**
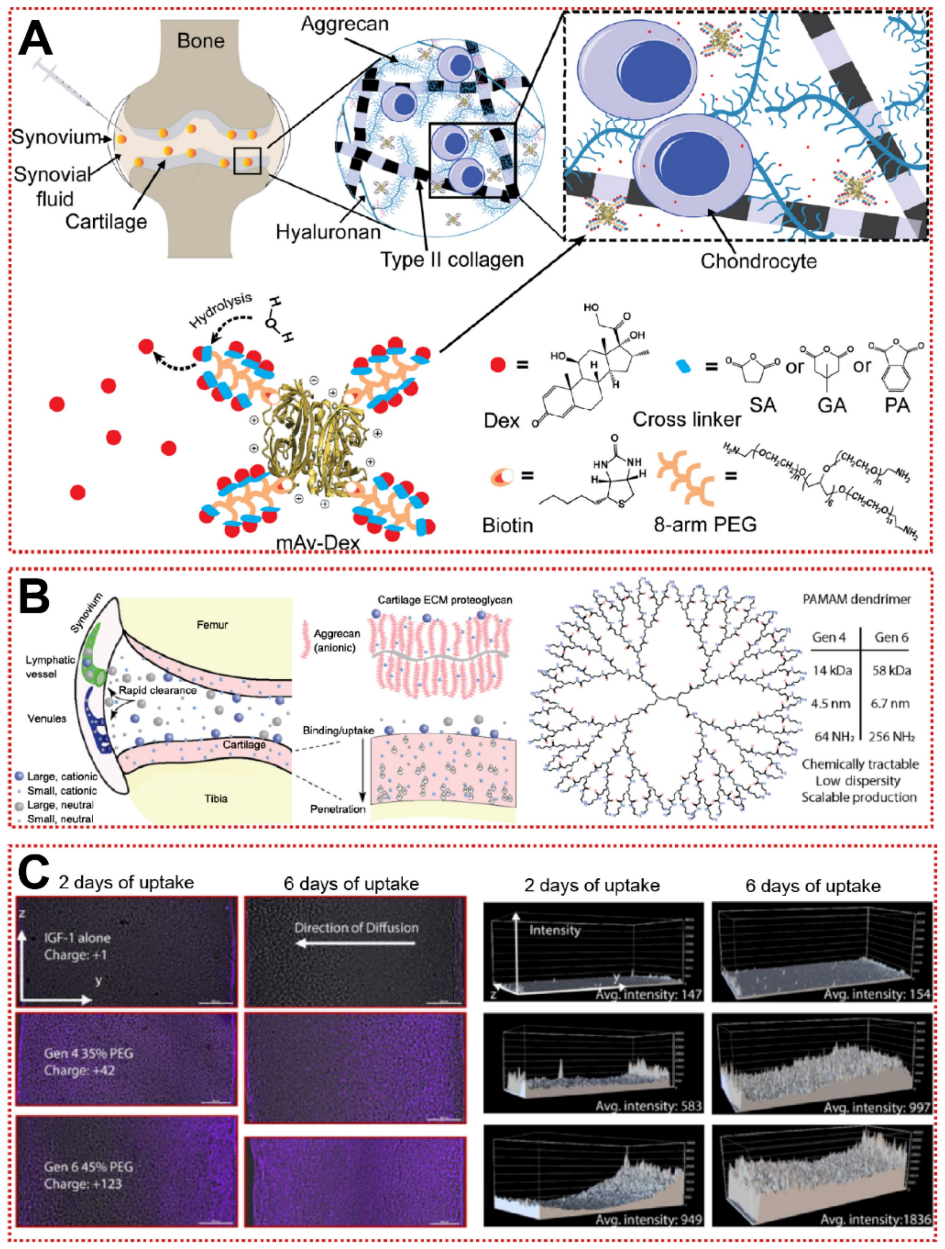
Passive targeting strategy for OA treatment: **(A)** Schematic of charge-based intra-cartilage drug delivery of the nano-construct multi-arm Avidin conjugated with a small molecule drug 132. Copyright 2020, Elsevier. **(B)** Schematic of drug and carrier fates within joints following intra-articular injection based on size and charge 133. Copyright 2018, AAAS. **(C)** Confocal microscopy images of IGF-1 (purple) diffusion in articular cartilage and quantitative analysis of fluorescence 133. Copyright 2018, AAAS.

In addition, the constituent monomers of Aggrecan contain a large number of negatively charged carboxyl residues and sulfate residues, allowing cationic biomaterials to be attracted to the joints by electrostatic forces 162. Utilizing this unique property, nanoparticles with a positive charge tend to target the ECM and increase the uptake of chondrocytes through electrostatic attraction 162. A well-designed green fluorescent protein (GFP) was used to probe the optimal range of positive charge 163. As the GFP surface charge increased from +9 to +36, the permeability of GFP in the cartilage layer gradually diminished and was replaced by a stronger cellular uptake rate 163. The +9 GFP can be efficiently taken up by chondrocytes and human knee joints, while the electrically neutral GFP is completely inaccessible to cartilage explants 163. Similarly, when using cationic peptides as carriers, the net charge of +14 exhibited the best cartilage diffusion ability, suggesting that cations need to be applied with attention to the optimal charge range 131. Bajpayee et al. assembled a multi-armed anti-biotin protein, succinic acid (SA), glutaric acid (GA) and phthalic anhydride (PA) as a +25 mV cationic nanocarrier (mAv-Dex) to deliver Dex (Figure 6A) 132. mAv-Dex effectively inhibited IL-1α-induced ECM loss and nitrite release within two weeks 132. Polyethyleneglycol-functionalized polyamidoamines (PAMAM) with hundreds of primary amine residues are ideal cationic carriers for the treatment of OA (Figure 6B) 133. PAMAM loaded with insulin-like growth factor 1 (IGF-1) penetrated into cartilage to an unprecedented depth of 1 mm and extended the retention time of IGF-1 to 30 days (Figure 6C) 133. Relative to the poor efficacy of free IGF-1, PAMAM-IGF-1 significantly alleviated osteophyte formation and aggrecan degradation in rats, significantly delaying the pathological changes of OA 133. Notably, since the interaction of synovial fluid with biomaterials in the articular cavity may lead to “off-targeting”, more efficient targeting strategies must be sought, rather than just staying with physicochemical properties 164.

### Proactive targeting strategy

#### Targeting cartilage and extracellular matrix

**Figure 7:**
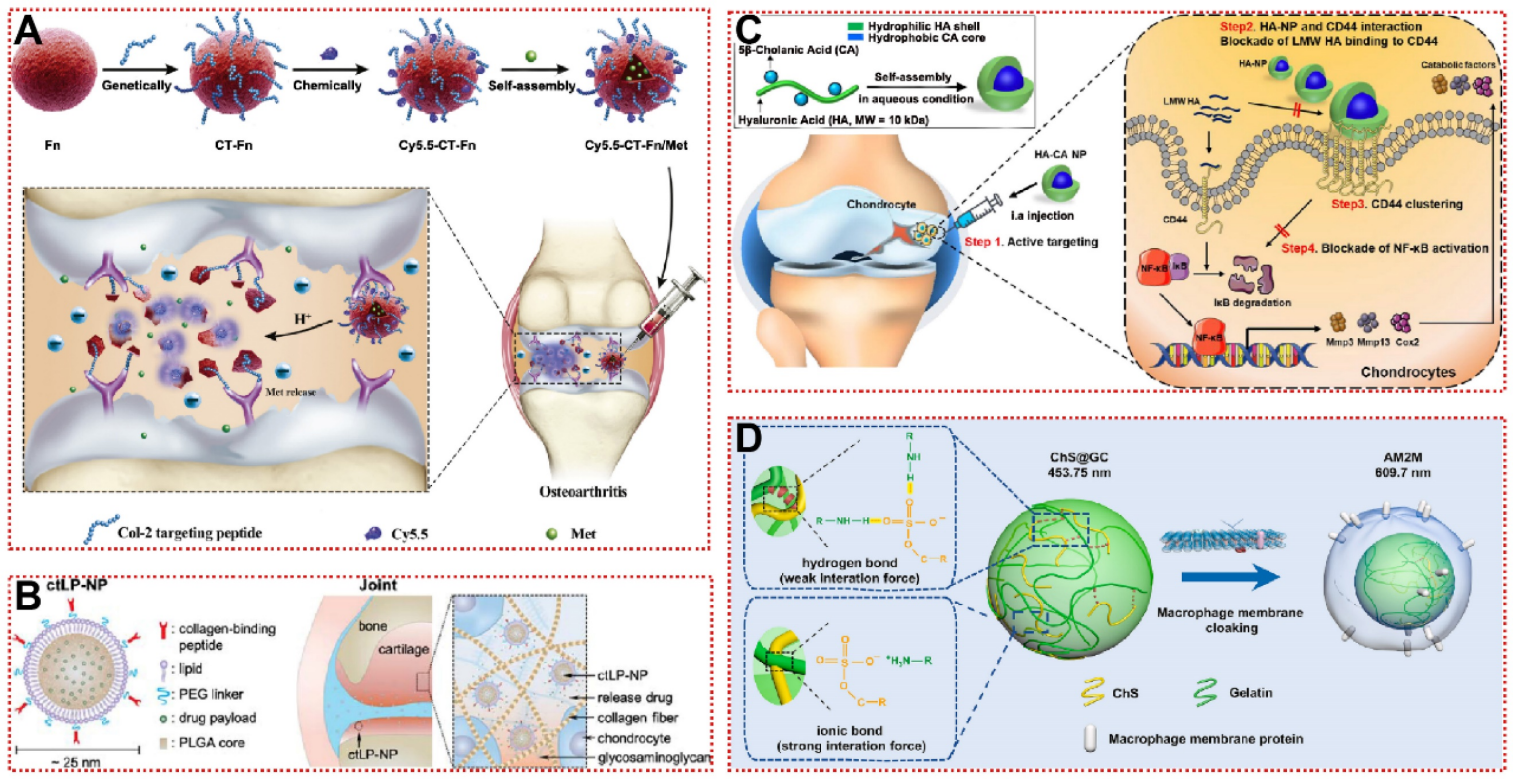
Cartilage targeting strategy for OA treatment: **(A)** Schematic illustration of synthesis, structure and anti-inflammatory mechanism of CT-Fn/Met drug delivery system in OA 23. Copyright 2020, Elsevier. **(B)** The design of collagen‐targeting ultrasmall lipid‐polymer hybrid nanoparticles (ctLP‐NPs) for targeted drug delivery to the joints 136. Copyright 2020, Wiley. **(C)** Schematic illustration of HA-NPs for treatment of OA 141. Copyright 2021, Elsevier. **(D)** Preparation procedure of macrophage membrane-cloaked ChS@GC (artificial M2 macrophage, AM2M) that loaded chondroitin sulfate (ChS) through multi-component ionic cross-linking and simple physical adsorption via ionic bond and hydrogen bond 143. Copyright 2021, Elsevier.

The identification of highly specific targeting ligands is often the paving stone for the study of active targeting strategies. In 2011 cartilage-targeting peptides (CAP, DWRVIIPPRPSA) screened by phage display technology have been shown to specifically induce the binding of nanoparticles to chondrocytes 165. They are not species-specific for *in vivo* studies in different model animals 165. Due to the unique three-dimensional structure and binding sites of short peptides, peptide-functionalized biomaterials have lower immunogenicity and higher operability 166. Ouyang et al. reported a cartilage-targeted gadolinium carbonate-PDA nanoparticle loaded with hesperidin (HGdPDW) that can be used for magnetic resonance imaging and OA therapy 134. CAP-modified HGdPDW has a homogeneous morphological structure and elemental distribution, accumulating abundantly in the cartilage of the lower femur and upper tibia *in vivo* 134. HGdPDW decreased Toll-like receptor 2 (TLR-2) expression on the plasma membrane surface, thereby downregulating NF-κB/Akt activation and reversing IL-1β-induced chondrocyte apoptosis 134. In the ACLT model, HGdPDW alleviated chondrogenic malformation and apoptosis marker caspase-3 protein production, revealing a potential mechanism for chondrocyte protection by HGdPDW 134. In addition to CAP, Rothenfluh et al. identified a type II collagen-targeting peptide (WYRGRL) that is now widely used in biomaterial modification 152. WYRGRL directs fluorescent probes into the cartilage matrix, so researchers can track articular cartilage lesion progression through *in vitro* live imaging 167. As shown in Figure 7A, Ho et al. attached WYRGRL to the surface of ferritin nanocages (CT-Fn) to overcome the drawbacks of conventional oral administration of metformin (Met) 23. The modified CT-Fn targeted type II collagen in articular cartilage, reducing cartilage macroscopic scores by 47% and 68% at weeks 2 and 6 23. Type II collagen-targeting peptide-modified liposomes also exhibited smooth penetration and prolonged retention in the mouse cartilage matrix (Figure 7B) 136. Based on the previous work, Lu et al. designed a functionalized nanoparticle (WPV-CuO) with MMP-2 cleavage peptide as a linker for dual-targeting peptides of type II collagen and MSC 137. WPV-CuO actively homed to cartilage and recruited MSC to cartilage differentiation *in vivo* 137. Transcriptome analysis revealed that phosphorylation activation of AKT was significantly downregulated in the WPV-CuO group, implying that the potential therapeutic mechanism of WPV-CuO is through inhibition of the PI3K/AKT/mTOR signaling pathway 137.

To further improve the active targeting of vectors, specific antibodies against articular cartilage have been incorporated into biomaterial fields of application. Compared with other methods, the antibody only binds and tightly attaches to the antigenic epitope on the surface of the corresponding antigen with high affinity and specificity, greatly improving the targeting of the vector 168. Type II collagen monoclonal antibody (MabCⅡ)-modified liposomes can be used for *in vivo* imaging by accumulating at the injured joint after intravenous injection 138. The amount of MabCⅡ binding correlated positively with the severity of OA, while no fluorescent signal was detected in healthy joints 139. Compared to the negative control, 124nm polymers coupled to MabCII (mAbCII-siNPs) achieved efficient siRNA chondrogenic delivery 140. mAbCII-siNPs not only silenced the expression of MMP13 and downstream related genes in chondrocytes, but also showed *in vivo* protection against ectopic calcification of synovial membrane, which was superior to steroid treatment 140. This suggests that specific antibody-based cartilage targeting strategies are worthy of further long-term study and promising for clinical trials.

For biocompatibility and adaptability considerations, some bionic biomaterials have also been applied for active targeting of OA 169, 170. The use of HA nanoparticles (HA-NP), rather than direct HA injection, allows targeted binding of highly expressed CD44 in damaged cartilage to competitively block activation of the NF-κB signaling pathway (Figure 7C) 141. HA-NP not only exhibited hyaluronidase resistance and cartilage penetration, but also dramatically alleviated destabilization of the medial meniscus (DMM)-induced cartilage destruction and subchondral bone plate thickening 141. The efficacy remained significant at intervals of up to 4 weeks of administration, suggesting that joint cavity injection of HA-NP may be a potential targeted therapeutic strategy for OA 141. Nanoghosts (NGs) made from mesenchymal stem cell membranes were reported to be a cell-free cartilage targeting platform, and the intracellular transport of NGs was analyzed by fluorescence co-localization techniques 142. Due to their inherent immunomodulatory and inflammatory homing ability, NGs were first endocytosed by chondrocytes and then entered the lysosome, subsequently downregulating the expression of COX2 and PEG2 at the mRNA and protein levels compared to the untreated group 142. Moreover, when using M2 macrophage membranes as a shell (Figure 7D), artificial macrophages (AM2M) block interleukin-induced acute inflammatory injury while avoiding chondroitin sulfate (ChS)-induced immune stimulation 143. AM2M has the greatest articular retention time and stable ChS release rate, successfully restoring the expression of MMP13 to normal levels 143. It is demonstrated that it is indeed feasible to mimic the secretion of anti-inflammatory cytokines by M2 macrophages in OA. For bionic biomaterials used in OA, there is a lack of sufficient systematic *in vivo* and *in vitro* studies to clarify the action mechanisms and long-lasting therapeutic effects, restricting their clinical translation. Overall, active targeting strategies based on cartilage and extracellular matrix are still in their infancy, further development of more desirable OA-targeting biomaterials can be achieved in the future by drawing on experience from other diseases such as cancer or immunomodulation.

#### Targeting synovium

Synovium is composed of macrophages, fibroblasts and vascular endothelial cells, which perform the functions of material exchange and secretion of synovial fluid 171. Apart from cartilage damage, synovial inflammation is also a major pathological characteristic of OA lesions 172, 173. Topical or systemic administration of corticosteroid is often used for synovial inflammation in OA, but their potential toxicity and excessive metabolism both make long-term use impossible and increase the risk of osteoporosis 174. For this reason, biomaterial therapeutic strategies targeting synovium have attracted interest. Initially, the researchers hoped to achieve synovial-targeted delivery by using RGD peptide binding to integrin αvβ3 on synovial vascular endothelial cells 144. However, the expression of integrin αvβ3 is not synovial specific and may lead to a non-specific accumulation of biomaterials extra-synovial 175, 176. Colombo et al. achieved targeted delivery of degradable nanoparticles (tBNPs-MTX) by coupling cyclic synovial homing peptides 145. This cyclic peptide showed strong binding capacity to von Willebrand Factor^+^ (VWF^+^) vascular endothelial cells and CD34^+^ hematopoietic stem cells in synovial tissue 145. tBNPs-MTX significantly reduced neovascularization density and leukocyte counts in rat synovium, further demonstrating the great potential of synovium as a therapeutic target for OA 145. In addition, a block polymer-based superoxide dismutase nanoparticle (SOD-NP) was reported to have a unique targeting ability to synovial fibroblasts, overcoming the excessive degradation of SOD 146.

**Figure 8:**
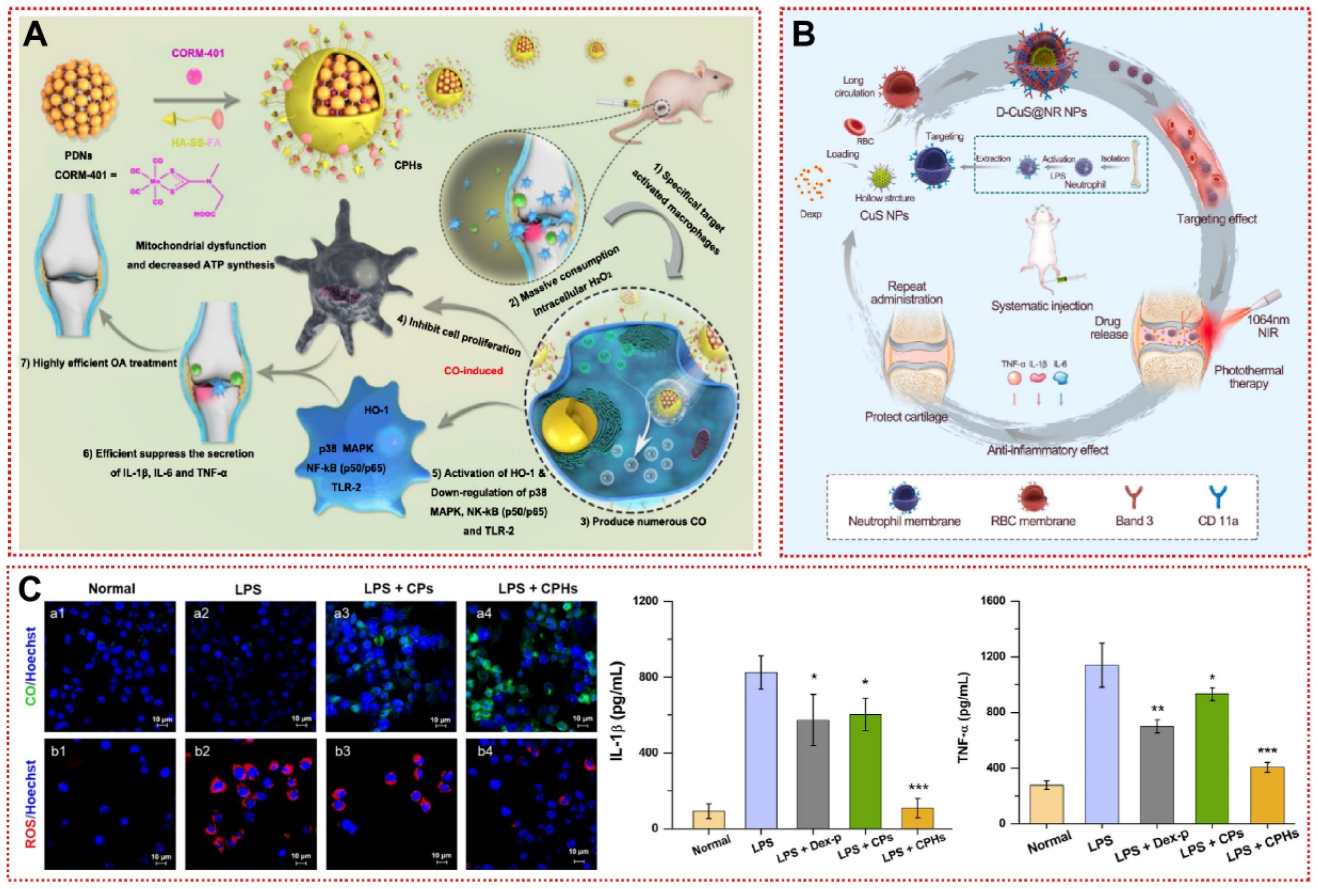
Synovial targeting strategy for OA treatment: **(A)** Schematic illustration for the preparation of CPHs as well as its related mechanisms for the effective treatment of osteoarthritis 150. Copyright 2020, Elsevier. **(B)** Changes in ROS/CO levels in activated macrophages and inhibition of inflammatory factor secretion after treatment with CPHs 150. Copyright 2020, Elsevier. **(C)** Schematic diagram of the preparation and therapy of the D-CuS@NR NPs 151. Copyright 2022, Elsevier.

In the inflammatory microenvironment, interferon-γ (IFN-γ) and TNF-α induce synovial macrophages to polarize toward the pro-inflammatory M1 type, leading to the release of downstream inflammatory factors IL-1β and IL-6 177. Modulation of the M1/M2 ratio of macrophages to suppress the progression of inflammation may be a promising intervention for OA 178. Zhou et al. grafted an anti-CD16/32 antibody onto a modified zeolitic imidazolate framework-8 (ZIF-8) to restrain hypoxia-inducible factor-1α (HIF-1α) and recover macrophage mitochondrial dysfunction 148. After macrophages were reprogrammed to M2 type, angiogenesis and inflammatory cell infiltration in the synovium were dramatically reduced, and ACLT-induced cartilage damage was reversed 148. More interestingly, Chen et al. designed and synthesized novel magnesium-based ligand containers (Rap-FA@MgDHIA) for the stepwise loading and release of Rap utilizing folic acid (FA) as a surface ligand 149. Folate receptor β (FRβ) is highly expressed on the membrane surface of M1 macrophages 149. Rap-FA@MgDHIA achieves targeting M1 type macrophages by binding to FRβ 149. Rap-FA@MgDHIA subsequently releases Rap to convert macrophages from a pro-inflammatory phenotype (M1) to an anti-inflammatory phenotype (M2) 149. After 14 days of Rap-FA@MgDHIA local injection, iNOS-positive M1-type macrophages in synovial tissue were remarkably minimized 149. Rap-FA@MgDHIA alleviated synovial inflammatory cell infiltration while articular cartilage and subchondral bone were successfully conserved 149. In another study targeting synovial macrophages, FA-modified nanoparticles (CPHs) acted as CO donors to inhibit MIA-induced articular damage in rats (Figure 8A) 150. CPHs induced apoptosis by depleting H_2_O_2_ in activated macrophages while decreasing mitochondrial membrane potential and ATP synthesis, yet showed a satisfactory safety profile for normal macrophages 150. Lower ROS levels and higher CO levels reduced the macrophage-mediated inflammatory response (Figure 8B) 150. Considering the potential uptake by macrophages and lack of targeting in the circulation by conventional drug delivery, Xue et al. fused erythrocyte membranes and neutrophil membranes on a hollow copper sulfide surface to provide a promising platform (D-CuS@NR NPs) for inflammation-associated diseases (Figure 8C) 151. The modification of the composite membrane not only non-specifically adsorbs a variety of serum inflammatory factors, but also rapidly accumulates in the articular cavity at 60 min to inhibit cartilage wear 151. D-CuS@NR NPs can release Dex while also responding to NIR to produce photothermal effects for adjunctive therapy, demonstrating a new mode of OA treatment 151. Additionally, self-assembled micellar nanoparticles (DS-TA NPs) of dextran sulfate-triamcinolone acetonide conjugates specifically target the scavenger receptor class A (SR-A) on the surface of activated macrophages to reduce the expression of pro-inflammatory cytokines for promising targeted therapy of OA 179. In summary, the engineering of targeted biomaterials for synovial cells optimizes the biodistribution and potency of drugs *in vivo* as a promising therapeutic strategy for OA.

#### Targeting subchondral bone

Although cartilage degeneration and ECM loss have long been considered the primary causative factors of OA, there is growing evidence that subchondral bone lesions precede the onset of cartilage loss 4. Bone trabeculae and platelets in the subchondral bone are arranged in an appropriate proportional distribution to disperse mechanical stress, and osteoclast-mediated bone resorption and osteoblast-mediated osteogenesis are counterbalanced 4. After the onset of the lesion, the over-activated osteoclasts of the subchondral bone induce the invasion of blood vessels and nerves into the cartilage layer, triggering inflammation and pain 180. Several preclinical studies have been conducted to show the effectiveness of OA treatment options targeting abnormal subchondral bone remodeling and angiogenesis 181-183. With only the simplest drug injections to discuss efficacy on subchondral bone, rather than specific delivery, there remains a potential risk of nonspecific distribution of the drug in the articular cavity or systemically. Bisphosphonates (BPs) are used as highly effective bone targeting agents to inhibit osteoclast activity, and biomaterials coupled to BPs are also used to carry other active molecules for basic bone-related therapies 82. BPs-modified nano-apatite (NP-BP) achieves targeting of subchondral bone by responding to lower pH in the microenvironment 82. Since the bone resorption of subchondral bone in the early stage of OA was inhibited by NP-BP, the pain behavior of rats was relieved and the three-dimensional structure of the cartilage layer was preserved intact within 90 days, demonstrating that targeting subchondral bone has favorable feasibility and prospect for the management of OA 82. In addition, targeting crystalline mineral loss and reduction in collagen mineralization in subchondral bone may also be a potential target for OA 184. Yet, treatment options that target subchondral bone present enormous difficulties due to the natural barrier of articular cartilage and the low blood circulation to the subchondral bone. Although there are limited studies on targeting subchondral bone, the future development of subchondral bone-targeting biomaterials based on surface modification and innovative structures will be a new direction for OA treatment.

## Limitations and challenges of clinical translation

Although biomaterials hold exciting promise for improving drug solubility and precision release, the potential risks and unknown chronic toxicity of these materials in living organisms limit their clinical translation. The size of most biomaterials reaches the nanometer dimension, which allows them to easily cross multiple biological barriers and be non-specifically endocytosed by immune cells 185. Although no visceral toxicity or other complications have been reported in any of the biomaterials discussed above, the risk of temporary organ accumulation, sudden release or accidental off-target remains is still a concern. In addition to focusing on recovery from OA lesions, researchers should have a sufficient long-term understanding of the normal physiological functions of joint tissue (e.g., mechanical strength, synovial fluid composition, and nutrient supply) to ensure the safety of biomaterials. The polymers synthesized in the experiments usually do not reach 100% polymerization, although the polymers themselves are not toxic, the residual polymer monomers may promote apoptosis or genotoxicity 186. Around 100 nm chitosan, the most common natural biopolymer in the biomedical field, would cause significant neurotoxicity, liver damage and reduced hatchability in zebrafish 187, 188. In addition, joints are one of the most important motor tissues in the body, implant particles from sportswear will activate the NF-κB signaling pathway in macrophages and induce bone mineral loss 189. Chronic inflammation due to prosthetic wear particles may present an unpleasant challenge for patients with advanced OA undergoing total joint arthroplasty 189. Also, *in vivo* evaluation of simulated biomaterials using *in vitro* stem cell models or bone like organs has recently received much attention, which would be more convincing than toxicity experiments with single cell lines 190, 191.

The biodistribution and toxicity of the delivery platforms within the joint will also be influenced by the strength of the surface charge. Positively charged cationic materials tend to have easier uptake by cells and are therefore often used for therapeutic RNA delivery 192. However, when the charge intensity or concentration is too high, either cation or anion, it will trigger mitochondria-mediated cellular autophagy and plasma membrane damage 193. In previous studies, cationic nanoparticles were considered as ideal carriers for targeting cartilage using electrostatic attraction. However, as the surface charge continues to rise, excessive electrostatic interactions will prevent the penetration of nanoparticles larger than +14 mV into deeper layers, while other non-covalent bonds (e.g. hydrogen bonds, hydrophobic bonds) formed between GAG and cationic peptides will also affect their permeability 131. On the other hand, for carriers applied for systemic injection, liposomes with high surface charge have increased hepatic accumulation and complement protein-mediated immune clearance relative to electroneutral liposomes 194. Researchers should be more cautious to examine whether the loss of GAG in the late stages of OA may lead to off-target effects of cation-targeting strategies and subsequently increase the cumulative toxicity in non-targeted tissues.

Due to the presence of synovial fluid, adsorption of synovial proteins on the surface of biomaterials will probably interfere responsive group breakage and exposure of targeting ligands 195. Although PEG modification can suppress protein adsorption and protein corona formation, it does not completely avoid albumin or complement protein binding 196. Brown et al. indicated that incubation in the synovial fluid would lead to an increase in cationic nanoparticle size and charge reversal 195. It is speculated that the protein corona formed by adsorbed synovial proteins may mask the functional ligands on the nanoparticle surface and subsequently affect their tissue transportation and responsive release within the joint. Hence, how to overcome the interference of synovial fluid with biomaterials to achieve efficient OA treatment will be a great challenge for clinical translation. They should be further tested in a diversity of joint tissues or in large animals to understand and confirm the efficacy of OA.

Finally, researchers should be cautious in their choice of model animals and means of inducing OA. Due to differences in species, age, and sex, none of the animal models can fully replicate all pathological processes in human OA 197. Researchers need to consider a combination of study objectives and concerns to determine the modeling approach. For example, spontaneous OA allows researchers to analyze the disease process from early to late stages in different time sequences. However, such OA models are time-consuming and expensive, while the variation between animals can be significant. Invasive surgery allows experimenters to achieve a less variable OA development and is currently the most commonly used and stable animal model of OA 198. Nevertheless, it is not suitable for studying the changes in OA during long-term wear and aging 198. Induction by chemical adjuvants such as collagenase can produce significant cartilage wear and pain perception in a short period of time 198. Yet, this is far from the pathogenesis of human OA. These different mechanisms of pathogenesis will perhaps influence the efficiency of drug targeting. Some models that produce large amounts of ROS or pH reduction rapidly may exhibit more pronounced responsive drug release relative to naturally occurring OA 199. Thus, a single model of OA treatment strategy cannot be fully equated with clinical efficacy. Future studies should include more convincing evidence to compare the efficiency of targeted release of drug-loaded biomaterials in different OA models.

## Conclusion

OA is a degenerative disease characterized by loss of articular cartilage and local inflammation that affects tens of millions of people worldwide. The unknown pathogenesis of OA leads to clinical treatment strategies that do not completely eradicate cartilage degradation. Conventional intra-articular injections or oral administration of drugs are often rapidly cleared by phagocytes or metabolic organs resulting in low drug utilization and a narrow therapeutic window. The application of targeted and responsive biomaterials in the biomedical field is bringing new light to the treatment of OA. Efficient drug delivery systems based on the OA microenvironment (enzymes, ROS, pH, stimulation) and lesion sites (cartilage, synovium, subchondral bone) have shown promising applications. Not only do they maximize the therapeutic potential of drug molecules, but more importantly they improve the biodistribution and delivery of drugs. The "bio-missile" based on biomaterials penetrates nanoscale pores and has long-lasting controlled release, avoiding the pain and economic burden of multiple high-dose administrations to patients. By modifying and optimizing the structure and physicochemical properties, biomaterials overcome the therapeutic bottleneck of drug monomers and enable the integration of treatment. The controlled release of drugs in combination with microenvironmental changes assists in better mastering the pathological stages of OA, while the coupling of targeted ligands opens new horizons for precision therapy. Moreover, these targeted and responsive biomaterials may be used for other OA-affected joint tissues in the future, including fat pads and joint ligaments.

Nevertheless, biomaterial-based clinical therapies for OA are still in their infancy, and there is a lack of sufficient clinical studies to demonstrate the efficacy and safety of these strategies. Previous studies have also shown that the selection of natural cartilage components based on hyaluronic acid and collagen as carriers may yield better therapeutic benefits and safety. Also, the cartilage layer is thicker in primates, which would greatly prolong the diffusion of biomaterials. These findings should be further validated in a more closely related human model (e.g., rhesus monkey). In addition, further validation is needed to determine if the effectiveness of these targeted ECM strategies is compromised in the late stages of OA when the collagen network of chondrocytes is depleted. On the other hand, new multifunctional biomaterials involve more complex and sophisticated technologies, often leading to expensive economic costs in clinical applications, which should be taken into account. Since the criteria for toxicity evaluation of biomaterials at the development stage are not yet standardized, the absence of a quality evaluation system will lengthen the cycle of the approval process, which is not conducive to the clinical translation of biomaterials. To break the existing barriers and accelerate the clinical translation of targeted and responsive biomaterials, we need to deepen our understanding of OA not only at the molecular and cellular levels, but also to speed up the research process of novel active drug molecules and the search for more specific drug carriers to achieve stronger delivery effects. To meet these challenges, broad and close collaboration and cross-disciplinary research among materials scientists, biologists and clinicians are required. Looking ahead, with the continuous improvement of regulatory mechanisms and experimental techniques, the targeted and responsive biomaterials are expected to become a panacea for OA patients.

## Figures and Tables

**Table 1:** Responsive biomaterials for osteoarthritis treatment

Materials	Responsive stimulus	Responsive components	Particle size	Drugs	Biological functions	References
MRC-PPL@PSO	MMP-13	Specific peptide substrate of MMP-13 (H2N-GPLGVRGC-SH)	121.5 ± 26.1 nm	Psoralidin	Resist chondrocyte inflammation and lower MMP-13 expression	22
CMFn@HCQ	MMP-13	Specific peptide substrate of MMP-13 (H2N-GPLGVRGC-SH)	22 nm	Hydroxychloroquine	Increase cartilage retention time and restore the complete structure of the ECM	73
TG-18 hydrogel	MMPs	Triglycerol monostearate (TG-18)	Not available	Triamcinolone acetonide	Release medication on demand and relieve paw swelling	74
Gelatin microspheres	Collagenase	Gelatin	10~30 μm	IL-4, IL-10 and IL-13	Decrease NO production in chondrocytes by 80% *in vitro*	75
PTKU@DEX	ROS	Polythioketal (PTK)	460 nm	Dexamethasone	Reverse the expression of iNOS and Arg-1 in synovial macrophages and prevent their polarization to M1 type.	76
TKCP@DEX	ROS	Thioketal (TK)	60 nm	Dexamethasone	Enrich Dex in articular cartilage to improve cartilage wear and reduce the expression of IL-6 and MMP-13	24
Dex-pPADN	ROS	Phenylboronic acid	89.6 ± 9.5 nm	Dexamethasone	Dramatically inhibit synovial neutrophil infiltration and restore aggrecan content in the cartilage layer	77
RRHMs	ROS	Fe^2+^ and sodium bicarbonate	344.2 ± 10.3 μm	Dexamethasone sodium phosphate	Release anti-inflammatory drugs via Fenton response to scavenge ROS and rescue collagen type X content	78
DEX@PPNP	ROS	Tetrahydroxydiboron (THDB)	190 nm	Dexamethasone	Suppress pain behavior and angiogenesis of subchondral bone in OA mice	79
AG@MSNs-PAA	pH	Polyacrylic acid (PAA)	120 nm	Andrographolide	Restore IL-1β-induced chondrocyte apoptosis and Col2α1 gene expression.	80
MOF@HA@PCA	pH	MIL-100 (Fe)	123.4 nm	Protocatechuic acid	Inhibit osteophyte formation, promote chondrocyte proliferation and articular cartilage regeneration in ACLT model	81
NP-BP	pH	Nano-apatite	400 nm	Bisphosphonates	Impair subchondral bone TRAP^+^ osteoclast bone resorption activity and angiogenesis	82
Rh-PLGA-NPs@NH_4_	pH	NH_4_HCO_3_	190.7 ± 1.2 nm	Rhein	Upregulate chondrocyte uptake and scavenge ROS *in vitro*	83
ACP	pH	Tertiary amine groups	170 nm	Curcumin	Recover articular cartilage ECM integrity by targeting enrichment in OA joints by responding to acidic environments	84
CSL@HMSNs-Cs	pH	Chitosan	260 nm	Celastrol	Restrain chondrocyte NF-κB signaling pathway activation and diminish the concentration of inflammatory factors and MMPs in the supernatant	85
F127/COS/KGN_DCF_	Thermo	Pluronic F-127	650 nm (4°C) and 305 nm (37°C)	Kartogenin and diclofenac	Minimize matrix loss of bone surface, sclerotic bone and microfracture on 14 weeks	86
pNIPAM+HA&DS	Thermo	Poly(N-isopropylacrylamide)	Not available	Hyaluronic acid and diclofenac sodium	Reverse OA synovial hyperplasia and promote GAG deposition to protect chondrocytes from degeneration	87
RB@MPMW	NIR	Polydopamine	114.1 nm	Rapamycin and bilirubin	Significantly reduce autophagy-related p65 phosphorylation and restore mitochondrial homeostasis	25
MoS_2_@CS@Dex	NIR	MoS_2_	78 ± 18 nm	Dexamethasone	Invoke activated macrophage apoptosis pathway and reverse mitochondrial depolarization in chondrocytes	88
PEG-MNPs	Magnetic field	Metallic spherical cobalt	30 nm	Not available	Accelerate transport in bovine articular cartilage by nearly 50-fold under the action of an alternating magnetic field	89
MC-MNCs	Magnetic field	Magnetic iron oxide	258 nm	Meloxicam	Reduce arthritic pain and loss of collagen in articular cartilage of rats	90
MTX-Au/Fe/Au	Magnetic field	Iron-shell nanoparticle	135 nm	Methotrexate	Accumulate at the site of arthritis and prolong the retention time to relieve synovial hyperplasia	91
AuSPION	Magnetic field	Magnetic iron oxide	10 ± 3 nm	Methotrexate	Reduce immunostaining positive cells for TNF-α and IL-1β in synovium	92
SPIONs	Magnetic field	Magnetic iron oxide	1 μm、10 μm	Dexamethasone	Uptake by synovial fibroblasts and increase residence time of Dex	93
PEI-SPIO/siRNA	Magnetic field	Magnetic iron oxide	10 nm	siRNA	Suppress macrophage activation and cartilage destruction at joint edges	94
CAG-MSCs scaffolds	Magnetic field	Magnetic iron oxide	Not available	Not available	Promote BMSC chondrogenic differentiation and restore collagen content at the site of cartilage defects in rabbits	95
US-MB	Ultrasound	Microbubbles	Not available	Diclofenac sodium	Suppress inflammation and neovascularization in peri-ankle tissues	96
iELPs	Ultrasound	Microbubbles	113.3 ± 4.6 nm	Methotrexate	Restore mean clinical indices and ankle disorders in arthritic mice	29
DexSP@LPs-PEG-FA	Ultrasound	Microbubbles	158 ± 1.4 nm	Dexamethasone	Increase the uptake of liposomes into activated macrophages and decrease the level of inflammatory cytokines in the blood	27
PFP-Dex@NDs-PEG-FA	Ultrasound	Perfluorocarbon nanodroplets	311.6 ± 3.8 nm	Dexamethasone	Down-regulate joint inflammatory cell infiltration and pannus formation	97
Rh/SPX-HSA	Ultrasound	Sparfloxacin	10 nm	Rhodium nanozyme	Produce oxygen to resist angiogenesis and generate reactive oxygen species to induce apoptosis in synovial fibroblasts	98

**Table 2:** Targeted biomaterials for osteoarthritis treatment

Materials	Targeted strategies	Targeted cells	Particle size	Drugs	Biological functions	References
AuNPs	Small size	Synoviocytes	5 nm	Not available	Reduced MMPs and LDH activity as well as HA concentration, but did not affect PGE2 levels	129
PEG-MnO_2_ NPs	Small size and cation	Chondrocytes	10.92 nm	Not available	Down-regulate the transcription of various antioxidant genes such as superoxide dismutase to baseline levels to restore normal chondrocyte function	130
CPC	Cationic peptide	Chondrocytes	Not available	Not available	Provide sufficient electrostatic driving force to deliver the carrier into the articular cartilage space without affecting chondrocyte viability	131
mAv-Dex	Cationic Avidin	Chondrocytes	7.6 nm	Dexamethasone	Lower IL-1α-induced GAG loss, chondrocyte death and ROS inflammatory response	132
Dendrimer-IGF-1	Cationic polyamidoamine	Chondrocytes	Not available	Insulin-like growth factor 1	Penetrate the entire layer of cartilage and prolong the residence time of IGF-1 to save cartilage from degeneration	133
HGdPDW	Cartilage affinity peptide (DWRVIIPPRPSA)	Chondrocytes	110 nm	Hesperetin	Down-regulate TLR-2/NF-κB/Akt phosphorylation activation to resist chondrocyte apoptosis and ECM loss	134
PLGA NPs	Type II collagen-targeting peptide (WYRGRL)	Chondrocytes	256 nm	Not available	Guide nanoparticles to bind tightly to chondrocytes for *in vitro* fluorescence imaging	135
CT-Fn/Met	Type II collagen-targeting peptide (WYRGRL)	Chondrocytes	20 nm	Metformin	Significantly reduce the number of MMP-13 positive cells, bone flab formation and cartilage surface lesions	23
ctLP-NPs (MK)	Type II collagen-targeting peptide (WYRGRL)	Chondrocytes	25 nm	MK-8722	Repair cartilage structural damage and depress TNF-α and IL-6 concentrations in OA femurs	136
WPV-CuO NPs	Type II collagen-targeting peptide (WYRGRL), MSC-targeting peptide (VTAMEPGQ), MMP-2 sensitive sequence (GALGLP)	Chondrocytes, MSCs	5 nm	Not available	Recruit MSCs to differentiate into chondrocytes and repress PI3K/AKT/mTOR signaling pathway to improve tissue damage	137
MAbCII liposome	Type II collagen antibodies	Chondrocytes	200 nm	Not available	Guide the tail vein injection of liposomes to selectively accumulate in the articular cartilage at the OA lesion, rather than in healthy cartilage	138
MAbCII liposome	Type II collagen antibodies	Chondrocytes	100 nm	Not available	Enhance joint aggregation with OA severity for *in vivo* real-time fluorescence imaging	139
MAbCII-siNPs	Type II collagen antibodies	Chondrocytes	124 nm	MMP-13 siRNA	Targeted silence MMP-13 expression in mice to protect against cartilage damage and prevent ectopic calcification of synovium	140
HA-NPs	Bindng to CD44	Chondrocytes	221 ± 1 nm	Not available	Reduce NK-κB promoter activity and block CD44-induced expression of MMP3, MMP13 and COX2	141
Nanoghosts	MSC membrane	Chondrocytes	200 nm	Not available	Alleviate cartilage tissue damage and reduce production of multiple pro-inflammatory cytokines	142
AM2M	Macrophage membrane	Chondrocytes	608.57 ± 11.37 nm	Chondroitin sulfate	Mimic physiological environment cytokine release to promote chondrocyte proliferation and restore articular cartilage GAG content	143
RGD-PEG-L	RGD peptide	Vascular endothelial cells	Not available	Dexamethasone sodium phosphate	Targeted deliver to blood vessels in areas of inflammation to enhance the efficacy of Dex-p	144
tBNPs-MTX	Synovial targeting peptide (CKSTHDRLC)	Synovial vascular endothelial cells	160 nm	Methotrexate	Disrupt synovial neovascularization by promoting CD34^+^ endothelial cell death	145
SOD-NPs	Permeable porous polymersomes	Synovial fibroblasts	120 nm	Superoxide dismutase	Reduce MMP-13 and Adamts-5 secretion after endocytosis by synovial fibroblasts to reduce synovitis	146
IL-1Ra NPs	IL-1 receptor antagonist	Synoviocytes	270 ± 5 nm	Not available	Extend the joint retention of IL-1Ra and specifically block IL-1β-induced NK-κB activation	147
ZIF-8 NPs	Anti-CD16/32 antibody	Macrophages	160 nm	S-methylisothiourea hemisulfate salt and catalase	Eliminate H_2_O_2_ and NO production in M1 macrophages and reprogram mitochondrial metabolism to facilitate M2 conversion	148
Rap-FA@MgDHIA	Folic acid	Macrophages	3 nm	Rapamycin	Promote the conversion of M1 macrophages to M2 macrophages to reduce the inflammatory cell infiltration of synovium and synovitis score	149
CPHs	Folic acid	Macrophages	270 ± 1 nm	CORM-401	Release CO to deplete intracellular H2O2 and downregulate LPS-induced cellular inflammation to protect cartilage ECM from degradation	150
D-CuS@NR NPs	Neutrophil-erythrocyte membrane	Macrophages	178 nm	Dexamethasone sodium phosphate	Extend the circulation time *in vivo* to adsorb plasma inflammatory factors and accumulate to the site of inflammation	151
DS-TA NPs	Binding to the scavenger receptor class A	Macrophages	70 nm	Triamcinolone acetonide	Reduce expression of IL-1β and IL-6 in articular cartilage and restore bone remodeling in subchondral bone	151

## Abbreviations

OA: osteoarthritis
NSAIDs: non-steroidal anti-inflammatory drugs
HA: hyaluronic acid
ROS: reactive oxygen species
ECM: extracellular matrix
GAG: glycosaminoglycan
MMPs: matrix metalloproteinases
IL-1β: interleukin-1β
NO: nitric oxide
PGE2: prostaglandin E2
TNF-α: tumor necrosis factor-α
IL-6: interleukin-6
ADAMTS: a disintegrin and metalloproteinase with thrombospondin motifs
CS: chondroitin sulfate
ACI: autologous chondrocyte implantation
MRC-PPL: poly(2-ethyl-2-oxazoline)-poly(ε-caprolactone)
PSO: psoralidin
TA: triamcinolone acetonide
O^2-^: superoxide anions
H_2_O_2_: hydrogen peroxide
OH·: hydroxyl radicals
NO·: nitric oxide radicals
^1^O_2_: singlet oxygen
GSH: glutathione
SOD: superoxide dismutase
PTK: polythioketal
HDI: hexamethylene diisocyanate
PTKU: polyurethane
Dex: dexamethasone
TK: thioketal
MIA: monosodium iodoacetate
HM: hollow microspheres
CO_2_: carbon dioxide
AG: andrographolide
PAA: polyacrylic acid
MOF: metal-organic frameworks
PCA: protocatechuic acid
NP-BP: bisphosphonate-conjugated nano-apatite
CSL: celastrol
PAE: poly(β-amino ester)
Rh: rhein
KGN: kartogenin
DCF: diclofenac
NIR: near-infrared
PTT: photothermal therapy
PDA: polydopamine
Rap: rapamycin
Br: bilirubin
ACLT: anterior cruciate ligament transection
GFP: green fluorescent protein
SA: succinic acid
GA: glutaric acid
PA: phthalic anhydride
PAMAM: polyamidoamines
IGF-1: insulin-like growth factor 1
CAP: cartilage-targeting peptides
TLR-2: toll-like receptor 2
Met: metformin
MabCⅡ: Type II collagen monoclonal antibody
DMM: destabilization of the medial meniscus
NGs: nanoghosts
ChS: chondroitin sulfate
VWF: von willebrand factor
SOD: superoxide dismutase
IFN-γ: interferon-γ
ZIF-8: zeolitic imidazolate framework-8
HIF-1α: hypoxia-inducible factor-1α
FA: folic acid
FRβ: folate receptor β
BPs: bisphosphonates
